# Acupuncture for migraine prophylaxis: A protocol for systematic review and Bayesian network meta-analysis

**DOI:** 10.1097/MD.0000000000032442

**Published:** 2022-12-23

**Authors:** Wenyan Zhu, Yiwen Cai, Yijun Zhan, Liaoyao Wang, Yue Wu, Jian Pei

**Affiliations:** a Department of Acupuncture, Longhua Hospital, Shanghai University of Traditional Chinese Medicine, Shanghai, China.

**Keywords:** acupuncture, Bayesian, migraine, network meta-analysis, protocol

## Abstract

Migraine causes health problems in 1 billion people worldwide and imposes a huge social burden. Acupuncture therapy has a good clinical effect in migraine prophylaxis and is recommended by authoritative journals. We plan to conduct a Bayesian network meta-analysis to compare the efficacies of different acupuncture therapies.

**Methods:**

We will search PubMed, Web of Science, Embase, Cochrane Library, China National Knowledge Infrastructure, VIP database for Chinese technical periodicals, Chinese biological medical database, WanFang Data, Cochrane register of controlled trials, Chinese Clinical Trial Register, and ClinicalTrials.gov from their inception to July 1, 2022, for randomized controlled trials that studied different acupuncture therapies and other therapies for the preventive treatment of migraine. Migraine episodes, migraine days, headache intensity, and adverse events will be counted as outcomes. Two reviewers will independently complete the study selection, data extraction, and risk of bias assessment of all filtered trials. Pairwise meta-analysis and Bayesian network meta-analysis will be performed (if applicable) through Review Manager 5.3 and the “gemtc” and “rjags” packages of the R software. Confidence in Network Meta-Analysis will be used to evaluate the quality and credibility of the evidence for each outcome.

**Results:**

The protocol will compare the efficacies of different acupuncture therapies for migraine prophylaxis.

**Conclusion:**

This study aims to help clinicians develop an effective and safe treatment plan for migraine prophylaxis.

## 1. Introduction

Migraine is a chronic primary headache that affects more than 1 billion people worldwide.^[1]^ As the primary parameter affecting the “years of life with disability” among people under 50 years of age,^[2]^ migraine brings a huge economic and social burden. Migraine is characterized by moderate or severe headache attacks with reversible neurological and systemic symptoms such as photophobia, phonophobia, nausea, and vomiting.^[3]^ With an in-depth study of the clinical features and continuous update of the diagnosis, migraine is viewed as a complex and variable neurological disorder, not just a vascular headache. The occurrence and development of migraines are related to genetic and environmental factors. Migraine is a complex neurological disease that involves multiple genes and factors. The pathophysiological mechanism of migraine is related to the theory of cortical spreading depolarization, vascular origin, and trigeminal neurovascular system.^[4]^ Accumulating evidence indicates the primary role for neuropeptides such as calcitonin gene-related peptide, pituitary adenylate cyclase-activating polypeptide, and glutamate are mediators of migraine and as important therapeutic targets.^[5–7]^ Migraine is an independent risk factor for stroke, and patients with migraine with aura have significantly higher rates of ischemic stroke, unstable angina, and transient ischemic attack than those without migraine^.[8,9]^ Psychiatric disorders such as depression and anxiety are highly prevalent in people with migraine, bringing substantial limitations in work, household work, and leisure/social activities^[10]^ and causing great distress to patients with migraine. Although numerous pharmacological treatments, including ß-blockers, non-steroidal anti-inflammatory drugs, ergotamine, antidepressants, calcium channel blockers, and anticonvulsants, are available,^[11–15]^ adverse events, such as gastrointestinal symptoms and central depression, that may be caused by drugs make patients hesitant to choose pharmacological treatment.^[16,17]^

Acupuncture, a non-pharmacological treatment in traditional Chinese medicine (TCM), has been used for thousands of years. Migraine is 1 of the predominant international diseases treated with acupuncture.^[18]^ With international authoritative journals affirming its effectiveness and safety in the treatment of migraine,^[19–21]^ acupuncture has increasingly been used as a complementary therapy for migraine prophylaxis. It is well tolerated with little risk of serious adverse effects. Acupuncture is a reasonable treatment option for patients who are unresponsive or intolerant of standard therapies. Neuroimaging confirmed that acupuncture can be integrated into pain-related brain regions through different neural afferent pathways that cause analgesic effects.^[22–24]^ Experimental studies have found that the mechanism of acupuncture for migraine involves inhibiting neurogenic inflammation, improving cerebral microcirculation,^[25]^ and regulating vasoactive substances.^[26,27]^ Animal experiments using a rat model of migraine have confirmed that electroacupuncture can inhibit hyperalgesia by decreasing the number of mast cells and macrophages and serum levels of inflammatory factors.^[28,29]^ Since 2000, the number of studies on acupuncture for migraine has increased yearly, and randomized controlled trials (RCTs) on acupuncture for migraine have been the most important research topic in this field.^[30]^ Acupuncture has been reported to be effective in reducing headache intensity, reducing the number of attacks, and has long-term effects.^[31–33]^ A clinical study showed that at the end of the 3-month treatment cycle, the proportion of participants who achieved ≥ 50% reduction in the number of migraine days was 62.8% in the manual acupuncture group and 42.9% in the naproxen group.^[34]^ Another study showed that at the end of a 4-week intervention, the frequency of migraine attacks decreased in the electroacupuncture group by 3.2% and in the sham acupuncture group by 2.1.^[31]^ Cochrane reviews compared acupuncture with drug prophylaxis, sham acupuncture, and placebo for migraine prevention. The not ineffective rate was significantly reduced by verum acupuncture compared to sham acupuncture (pooled RR 0.24; 95% confidence intervals 0.15–0.38).^[35]^ Acupuncture is as effective as conventional migraine prophylaxis treatments, such as valproic acid, topiramate, metoprolol, and flunarizine.^[36]^ In 2016, a Cochrane review suggested that acupuncture reduces migraine attacks more effectively than prophylactic drug treatment.^[37]^ Guidelines for acupuncture on mi0graine recommend various therapies, including manual acupuncture, electroacupuncture, and scalp acupuncture.^[38–40]^ Previous meta-analyses tended to classify different acupuncture therapies into 1 category and compared them with medicine or placebo, ignoring the characteristics of different acupuncture therapies and lacking comparison between them. Therefore, making the best choice for clinicians is inconvenient.

Bayesian network meta-analysis (NMA) is a new type of meta-analysis method that has been developed based on Bayesian statistics in recent years. NMA allows for the estimation of heterogeneity in the effect of any given treatment and inconsistency in the evidence from different pairs of treatments.^[41]^ We plan to conduct an NMA and compare the efficacy of different acupuncture therapies for migraine prophylaxis to obtain the potential optimal option among different treatments.

## 2. Methods

We will perform a systematic review and NMA in accordance with the preferred reporting items for systematic reviews and meta-analyses protocols (PRISMA-P) statement and the Checklist of Items to Include When Reporting a Systematic Review Involving NMA (PRISMA-NMA).^[42,43]^ The protocol was registered in PROSPERO (ID: CRD42022348029).

### 2.1. Criteria for considering studies in this review

#### 2.1.1. Types of studies.

Only RCTs will be included. The languages, regions, and publication statuses are not limited. Full-text articles should be available and contain sufficient data for the meta-analysis. Reviews, meta-analyses, conference papers, animal studies, basic research, duplicate publications, and RCTs designed to intervene in acute migraine attacks will be excluded. Any study with fewer than 10 participants will be excluded due to potentially high risk of publication bias and inflated magnitude of odds ratio to ensure the credibility of the findings.

#### 2.1.2. Types of participants.

Participants will include patients aged > 18 years who were diagnosed with migraine in accordance with the International Classification of Headache Disorders developed by the International Headache Society. Sample characteristics (e.g., age, sex, country, type of migraine, and course of disease) between the control and treatment groups should be consistent.

#### 2.1.3. Types of interventions.

In this article, the included RCTs should contain at least 1 type of acupuncture therapy as an intervention; acupuncture can be used as an intervention alone, or in combination with pharmacotherapy for migraine prevention or placebo. Acupuncture therapy can be related to acupoint stimulation, guided by TCM. The following treatments will be considered: manual acupuncture, electroacupuncture, heat needing, warm needling, dry needle, scalp acupuncture, auricular acupuncture, acupressure, bloodletting, and moxibustion. Other therapies not guided by TCM and not involving the use of acupoints will not be recognized as acupuncture therapies in this study. Pharmacotherapy for migraine prevention will be classified according to the ingredients.

#### 2.1.4. Types of control groups.

Control groups will include other acupuncture therapies, pharmacotherapy for migraine prevention, placebo, sham acupuncture, and a combination of any of the above methods. Trials that compared the 2 acupoint selections will be excluded.

#### 2.1.5. Types of outcome measures.

Our aim is to evaluate the effectiveness of acupuncture for migraine prophylaxis, that is, whether migraine attacks were reduced after acupuncture intervention. Thus, the primary outcome will be migraine episodes (the number of migraine attacks per month at the completion of the intervention). The following observations after treatment will be secondary outcomes: migraine days, headache intensity (measured using the Visual Analog Scale or other scales), and adverse events. If some related information such as the Headache Impact Test, migraine disability assessment, sleep quality assessment, and mood scale show the potential value and far-reaching significance of statistical analysis, they will be analyzed in the future.

### 2.2. Strategy for searching studies

Comprehensive retrieval will be performed in PubMed, Web of Science, Embase, Cochrane Library, China national knowledge infrastructure, VIP database for Chinese technical periodicals, Chinese biological medical database, and WanFang Data from their inception to July 1, 2022. No language and publication status restriction will be set in the search. In addition, the reference lists of the retrieved articles will be retrospectively reviewed to identify potentially eligible RCTs. The following keywords or Mesh terms in combination will be used in search strategy: “randomized controlled trial” “migraine” “headache” “acupuncture” “acupuncture therapy” “scalp acupuncture” “moxibustion” etc. We will use different retrieval strategies in accordance with the characteristics of different databases. The detailed search strategy for PubMed is shown in Table 1. We will search the Cochrane register of controlled trials, the Chinese clinical trial register, and ClinicalTrials.gov to find unpublished RCTs. Academic dissertations, research conference proceedings, and gray literature will be searched to reduce publication bias in our data. Furthermore, we will hand-search Google Scholar (https://scholar.google.com) and Baidu Scholar (https://xueshu.baidu.com) for relevant trials that may be missed while searching databases.

**Table 1:** Search strategy for PubMed.

Order	Search items
#1	Acupuncture [MeSH]
#2	Acupuncture therapy [Title/Abstract] OR electroacupuncture [Title/Abstract] OR manual acupuncture [Title/Abstract] OR heat needing [Title/Abstract] OR warm needling [Title/Abstract] OR dry needle [Title/Abstract] OR scalp acupuncture [Title/Abstract] OR auricular acupuncture [Title/Abstract] OR acupressure [Title/Abstract] OR bloodletting [Title/Abstract] OR moxibustion [Title/Abstract] OR acupoint [Title/Abstract]
#3	#1 OR #2
#4	Migraine Disorders [MeSH]
#5	Migraine [Title/Abstract] OR headache [Title/Abstract]
#6	#4 OR #5
#7	Randomized Controlled Trial [Publication Type]
#8	Randomized [Title/Abstract] OR placebo [Title/Abstract] OR randomly [Title/Abstract] OR clinical trials [Title/Abstract])
#9	#7 OR #8
#10	#3 AND #6 AND #9

### 2.3. Study selection and data extraction

**Figure 1.**
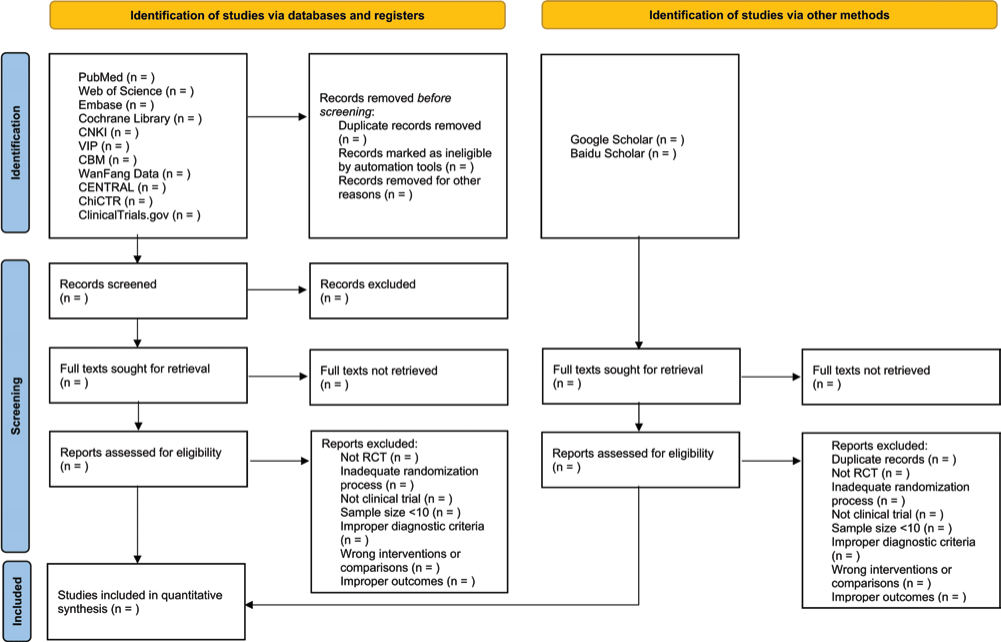
PRISMA flow diagram of selection process. PRISMA = preferred reporting items for systematic reviews and meta-analyses.

Study selection and data extraction will be performed by 2 reviewers independently, following the PICOS principle: participants, interventions, controls, outcomes, and study design. In cases of disagreement, a third reviewer will be consulted for assistance. During article screening, the title and abstract will first be read, and after excluding evidently irrelevant articles, full-text articles will be read to determine inclusion. Missing data should be supplemented by contact with the authors. If the reviewers cannot obtain key information, the document will be excluded. The specific selection process is illustrated in Figure 1 in the form of a PRISMA flow diagram.

Two reviewers will independently extract data from the eligible studies using standardized data extraction forms. After the data are extracted, the 2 reviewers will conduct a cross-checking. In case of disagreement, a third reviewer will be consulted to assist in the judgment. The following information will be included: basic information: title, first author, publication year, and country; trial design: randomization, blinding, allocation concealment; baseline characteristics of participants: sample size, sex, age, and migraine type; intervention: name of specific intervention, course of treatment, drug dosage; and outcome measures. For multi-arm studies comparing different types of acupuncture interventions, data will be extracted from all the relevant arms.

### 2.4. Assessment of risk of bias in included studies

Two reviewers will evaluate the risk of bias of all filtered trials using version 2 of the Cochrane risk-of-bias tool for randomized trials, and a third reviewer will settle disputes. For each trial, the randomization process; allocation concealment; blinding of participants and personnel; blinding of outcome assessment; missing outcome data; selective reporting; and other biases will be evaluated. The risk of bias of the trial will be rated as high, unclear, or low, in accordance with each domain.

### 2.5. Statistical analysis

#### 2.5.1. Pairwise meta-analysis.

Review Manager 5.3 (Cochrane Collaboration, http://tech.cochrane.org/home) will be used to perform the pairwise meta-analysis. A random-effects model will be used to estimate the effect size. We will estimate the summary effect size by using odds ratio for dichotomous variables and the mean difference for continuous variables when studies reporting outcomes used the same measurement. If headache tension is assessed using different scales, the standardized mean difference will be used. The 95% confidence intervals will be used to indicate whether the effect index was statistically significant. The extent of between-trial heterogeneity will be assessed the *I*^2^ test, with values over 50% indicating considerable heterogeneity.^[44]^

#### 2.5.2. Network meta-analysis.

We will use the network plot generated by Stata V.15.1 to compare multiple interventions simultaneously. An intervention is represented by a node and each line between 2 nodes represents a direct comparison between the 2 interventions. The node and line sizes are proportional to the number of included studies. Studies not connected to the network will be excluded from the NMA. We will use both random-effects and fixed-effects models for the NMA. The selection of the appropriate model will be based on the deviance information criterion; the model with the lowest deviance information criterion will be selected (with a difference > 5 indicating a significant difference in fit).^[45]^ We will perform NMA using a Bayesian framework via the “gemtc” package and “rjags” package of the R software (V.4.0.4). The specific parameter details are as follows. The initial value will be set to 2.5. Four chains will be built by the Markov Chain Monte Carlo method for the simulation that is iterated 100,000 times, of which the first 50,000 are annealed to remove the effect of the initial value. Potential scale reduced factor will be used to represent the convergence of the included studies. A potential scale reduced factor value close to or equal to 1 indicates a stable model, and the next step of data analysis can be carried out. Clinical and methodological heterogeneity will be evaluated by checking the characteristics and design of the included studies. Statistical heterogeneity in the network will be assessed quantitatively using the *I*² statistic, *I*^2^ > 50% indicating substantial heterogeneity. When a closed loop is present, a node-split model will be used to test for local inconsistency; values of *P* > .05 indicate good consistency; global inconsistency will be assessed by running the design-by-treatment interaction model.^[46]^ As there is no universal statistical method to analyze transitivity and similarity,^[47]^ these will be examined on account of clinical and methodological characteristics (experimental design, participant characteristics, outcome measures, and study quality, among others). We will show the results of direct, indirect, and pooled comparisons using forest plots. Finally, we will draw a ranking probability map to evaluate the efficacy and safety of each intervention comprehensively.

### 2.6. Subgroup analysis and meta-regression

If significant heterogeneity and inconsistency are found, possible sources will be explored. We will perform a meta-regression for pairwise meta-analysis and network meta-regression for NMA to examine the influence of potential effect modifiers. If sufficient number of studies is available, subgroup analyses will be conducted using effect modifiers as possible sources of inconsistency and/or heterogeneity. Potential effect modifiers could be (but will not be limited to) the duration of migraine, mean age of participants, pain intensity at baseline, treatment frequency, treatment sessions, and risk of bias. If there is insufficient data for subgroup analysis, we will provide a narrative summary.

### 2.7. Sensitivity analysis

The essence of NMA is to make indirect comparisons. To obtain a stable conclusion, a sensitivity analysis will be conducted to address whether the primary decision made in the review process is dominated by 1 or several studies. Several factors, such as a high risk of bias, specific population, sample size, methodological weaknesses, specific effect modifiers, and other factors that may affect the main results, will be considered. Relevant trials will be excluded to test the robustness of the study results.

### 2.8. Publication bias.

Funnel plots produced by Stata V.15.1. will be selected to assess the publication bias of NMA. Taking the effect size and sample size as coordinates, colors distinguish different interventions for comparison. If all studies are stacked around the centreline of the funnel plots, there is no publication bias, and asymmetric distributions indicate that there is publication bias.

### 2.9. Evaluation of the quality of the evidence

We plan to evaluate the quality and credibility of the evidence for each outcome using the confidence in network meta-analysis web application.^[48]^ Two reviewers will independently assess the following 6 domains: within-study bias; reporting bias; indirectness; imprecision; heterogeneity; and incoherence. Disagreements will be resolved through discussion or consultation with a third reviewer. The quality of evidence will be ranged from high, moderate to low, and very low.

### 2.10. Ethics and dissemination

This is a protocol for meta-analysis with no confidential personal data to be collected, and ethical approval can be skipped. The results will be disseminated in peer-reviewed publications.

## 3. Discussion

Although many options for migraine treatment are available, guidelines are updated in real time, and some new drugs, such as calcitonin gene-related peptide monoclonal antibodies and Gepants, have provided new choices for migraine treatment,^[49,50]^ about 10% to 15 % of patients with migraine have unsatisfactory drug treatment effects or poor compliance due to intolerance to adverse drug reactions,^[51]^ and 3% of migraines are converted to chronic migraine due to drug abuse or unclear diagnosis each year.^[52]^ A recent study showed that the use of non-steroidal anti-inflammatory drugs in the early stages of acute pain can lead to prolonged pain despite their short-term analgesic effects.^[53]^ After successful treatment with triptan during an acute migraine attack (meaning free from pain for 2 hours after treatment), the headache may recur within 24 hours or 48 hours.^[54]^ Therefore, preventive treatment for migraines is important. However, migraine prophylaxis still faces difficulties. Flunarizine is recommended as a prophylactic drug in migraine treatment guidelines in several countries,^[13–15]^ and some serious adverse events, such as depression and extrapyramidal symptoms, cannot be ignored.^[17]^ Identifying safe and effective migraine prevention therapies is a priority to reverse this situation. Acupuncture for migraine prophylaxis is also recommended by several guidelines,^[38–40,55]^ and relevant mechanism research and clinical research have always been hot topics in the field of pain. In addition to ordinary acupuncture, acupuncture therapy includes many different forms, such as electroacupuncture, moxibustion, and fire needling. In clinical practice, diverse acupuncture therapies are available for migraine prophylaxis, but the appropriate selection of an individual’s course of treatment has not yet been standardized. Clinicians often use a combination of several acupuncture therapies to determine the best treatment, which increases the burden of time and financial investment on patients. Most previous clinical trials compared a single acupuncture therapy with drug or sham acupuncture, and the effects of different acupuncture therapies have been poorly investigated. NMA, a technique that integrates direct and indirect comparisons across a set of multiple variables, can be used to analyze and rank multiple interventions in a single analysis. This study is the first Bayesian network meta-analysis protocol to compare various acupuncture therapies in the management of migraine prophylaxis, and we expect that our results will provide priority treatment options for acupuncture for migraine prevention.

This study may show some limitations. Given that China is the birthplace of acupuncture and has strong support for acupuncture therapy, the included studies may have geographical restrictions. The protocol is underway, and its results will be published with credible evidence to help clinicians choose the appropriate therapy for migraine prophylaxis.

## Acknowledgments

We thank all researchers in the field of acupuncture for migraine, who provided theoretical support for our research and sources of follow-up data.

## Author contributions

**Writing – original draft:** Wenyan Zhu, Yiwen Cai, Yijun Zhan, Liaoyao Wang, Yue Wu, Jian Pei.

**Writing – review & editing:** Wenyan Zhu, Yiwen Cai.
